# Therapeutic Notes

**Published:** 1896-10-24

**Authors:** 


### Therapeutic Notes.

### OVARIIN.

It was quite inevitable that to the long list of animal juices used in the new theraphy some preparation of ovariin tissue should be added. Up till the present time two classes of disease have been treated. Such preparations—firstly, the chlorosis, which is associated with some sort of uterine disorder; and, secondly, the various symptoms which follow in the train of amenorrhœa. Dr. Richard Mond has made a series of experiments on women suffering from amenorrhœa; in all eleven cases were treated, nine of them being the subject of this condition from various pathological causes, and two of them having been deprived of the uterus and its appendages by surgical operation. The symptoms of which the patients complained before being put on the treatment were for the most flushing of the head and profuse perspiration, feeling of pressure at the back of the head, headache, palpitation of the heart, sleeplessness, weakness in the limbs, and a general sense of depression. The symptoms appear to have improved considerably without any disagreeable effect; and Dr. Mond appears to have a high opinion of the treatment, which consists in giving one tabloid of ovariin four or five times a day; he used the tabloids supplied by

Merk, of Darmstadt. With regard to the treatment of chlorosis by the same means, Drs. Spillmann (*Rev. d. Therapeut.*, Sept. 1st, 1896), and Etienne have also made a series of experiments. They presume that this form of anæmia is associated, and perhaps dependent on disorders of the ovarian function, combined with irregular menstruation. They believe that the ovaries are glands—(1) With an external secretion; (2) which can eradicate organic toxines from the system; and (3) which have an internal secretion like the testes, and which plays an important part in the processes of nutrition. When the ovaries fail to perform their natural function one of the consequences is chlorosis. This may be due to cessation by the internal secretion in this, and so the administraion of ovarian extracts should modify the symptoms. According to these observers out of the six cases experimented upon three gave clear signs of improvement, with increased number of bed corpuscles, and increased percentage of hæmoglobin, with re-establishment of the menstrual function. In two cases a distinct reaction in temperature and pulse was noted, the temperature rising one or two degrees, and the pulse increasing in frequency. In these cases, in contradistinction to those of Dr. Mond, the prepared tabloids were not used, but the fresh ovaries from sheep were employed, the extract being obtained in the same manner as spermine is obtained in the Brown-Sèquard and D'Arsonnal method. The same authors also recommend the dried ovarian substance as suitable for the purpose.

### TANNALBIN.

The want of some astringent preparation which, without damaging the coats of the stomach, should exercise its styptic action on the mucous membrane of the intestines led to the preparation and manufacture of tannegen by H. Meyer. This substance, as is well known, is insoluble in acid media, and hence inoperative in the stomach; on the other hand, it readily dissolves in alkaline fluids and in the juice of the intestinal tract. But in its practical employment it has been recognised that most of astringent action is called into play in the first part of the small intestine when it first comes in contact with alkaline fluids. In chronic diarrhœa the seat of the disease is more often in the lower portions of the small intestine and in the various sections of the ascending, transverse, and descending colon, and hence, where its action is least inoperative, its presence is most required. In seeking for some chemical body which would continue to exercise its astringent properties throughout the length of the intestinal tract, Dr. Gottlieb (*Deuch. Med. Wocher*, No. 11, 1896) availed himself of the well-known physiological fact that albumin when submitted for a long time to a very high temperature presented a coagulum which was very resistant to the digestive juices of the stomach and alimentary tract. Such a form of albumen was combined with tannin, and, after submission to a high tempature, was found to defy the digestive ferments in the same manner as the simple albumen; it does, however, become slowly dissolved after prolonged exposure to these fermentations, and in so doing the tannin is set free from its combination. This combination of tannin and albumen has been manufactured by Knoll and Co., and it goes by the name of Tannalbin; it is quite insoluble in water, and should be taken in the form of powder, suspended in water or milk; eight grains four times a day appears a satisfactory dose; it makes no difference whether taken before or after meals. To Professor Vierardt (*Deuch. Med. Wocher*, No. 25, 1896) we owe most of the information we at present possess regarding its clinical use. He made trial of it both in the case of adults and of children, and altogether employed it in thirty cases, the majority of them presenting symptoms of chronic or tubercule intestinal catarrh. In all the cases amelioration of the symptoms was noticed with cessation of the diarrhœa. No symptoms of a disagreeable nature, and due to the drug, were recorded.

